# Cytomegalovirus (CMV)-specific cytotoxic T lymphocyte therapy resolve CMV diseases and refractory CMV infections in paediatric recipients of allogeneic haematopoietic stem cell transplantation

**DOI:** 10.1038/s41409-021-01499-0

**Published:** 2021-10-20

**Authors:** Xiaodong Wang, Uet Yu, Chunlan Yang, Chunjing Wang, Xiaoling Zhang, Yue Li, Changgang Li, Feiqiu Wen, Sixi Liu

**Affiliations:** Department of Hematology and Oncology, Shenzhen Children’s Hospital, Shenzhen, China

**Keywords:** Paediatrics, Therapeutics

**To the Editor:**

The use of CMV-specific cytotoxic T lymphocytes (CMV-CTLs) has shown advantages in controlling refractory and late CMV infection when combined with conventional antiviral treatment in haematopoietic stem cell transplantation (HSCT) recipients [1, 2]. However, the efficacy and safety of using CMV-CTLs in paediatric patients have not been fully evaluated.

A total of 307 paediatric patients received allogeneic HSCT at Shenzhen Children’s Hospital (SZCH), China between May 2018 and September 2020. The rate of CMV-DNAemia for all patients was 40.06% (123 patients). However, only nine patients were diagnosed with CMV end-organ diseases based on published diagnostic criteria [3]. All patients with CMV diseases, together with another patient with refractory CMV infections, were given CMV-CTL therapy in addition to standard antiviral treatment. The analysis of data was conducted with approval from the Institutional Review Board of SZCH. Written consents were obtained from parents or guardians for the analysis and publication of patient data.

PepTivator CMV pp65 peptide stimulated donor-derived CMV-CTLs were generated according to a previously published protocol [4]. The quality of CMV-CTLs was assessed by examining the expressions of IFN-γ and the activation marker NKG2D by CD8^+^ T cells using flow cytometry. The percentage of IFN-γ-producing T cells (CD3^+^IFN-γ^+^ cells) at each dose varied between individuals (ranging from 1.00% to 20.60% of total CD3^+^ T cells). However, it was confirmed that these cells were mainly CD8^+^ T cells (90.60–99.00% of total CD3^+^IFN-γ^+^ cells) and most of them expressed NKG2D (88.90–98.90% of the total CD3^+^IFN-γ^+^ cells).

The clinical characteristics of the patients are summarised in Table 1. Five boys and five girls aged 1–7 years (median 5 years) participated in this study. Primary diseases included thalassaemia and severe aplastic anaemia in 8 and 2 patients, respectively. All except one patient were recipients of haploidentical donors. All patients received myeloablative regimens comprising cyclophosphamide/busulfan/fludarabine/ thiotepa. Anti-thymocyte globulin (ATG) was used as the in vivo T cell depletion strategy. Graft-versus-host disease (GVHD) prophylaxis consisted of tacrolimus or cyclosporine in combination with mycophnolate mofetile and low-dose methotrexate, with or without post-transplantation cyclophosphamide.

**Table 1:** Patient characteristics.

Patient No	Sex	Age (years)	Diagnosis	Donor type	CMV serostatus	Time to CMV reactivation after HSCT	CMV infection/ disease	Peak CMV DNA titre (IU/mL)	Co-infections	aGVHD	cGVHD	T cell depletion strategy	GVHD prophylaxis	Chimerism	CMV antiviral treatment	Duration from CMV infection to CMV-CTLs therapy	Outcomes
1	M	7	TM	Haplo (father)	D + /R+	39 days	CMV pneumonia + retinitis	1.84 × 10^4^ (plasma)	HBV	-	Liver	ATG	PTCY + FK506 + MMF + LDMTX	40–99% for the first 4 months then >99% after 5 months.	GCV + FOS + CMV-IVIG	6 months	-Resolution of CMV DNAemia in 4 weeks. -Complete resolution of pneumonia in 2 weeks. -Blindness caused by CMV retinitis.
2	F	7	TM	Haplo (father)	D + /R−	63 days	CMV pneumonia	6.78 × 10^2^ (plasma)	PVB19, EBV DNAemia	-	Skin and liver	ATG	PTCY + FK506 + MMF + LDMTX	>95%	GCV + FOS	29 days	-Resolution of CMV DNAemia in 4 weeks. -Complete resolution of pneumonia in 2 weeks.
3	M	7	TM	Haplo (sister)	D + /R−	79 days	CMV pneumonia	1.44 × 10^3^ (BAL fluid), negative plasma CMV DNA	-	-	-	ATG	PTCY + FK506 + MMF + LDMTX	>99%	GCV + FOS + CMV-IVIG	13 days	-Resolution of CMV DNAemia in 3 weeks. -Complete resolution of pneumonia in 12 weeks.
4	F	3	TM	MSD (brother)	D + /R+	28 days	CMV pneumonia	2.26 × 10^4^ (BAL fluid), 4.16 × 10^3^ (plasma)	RSV	-	-	ATG	CSA + MMF + LDMTX	>99%	GCV	12 days	-Resolution of CMV DNAemia in 3 weeks. -Complete resolution of pneumonia in 4 weeks.
5	M	4	TM	Haplo (father)	D + /R−	48 days	CMV pneumonia	7.53 × 10^2^ (plasma), 7.70 × 10^2^ (BAL fluid)	EBV DNAemia, bacterial enteritis	-	-	ATG	PTCY + FK506 + MMF + LDMTX	>99%	GCV + FOS + CMV-IVIG	19 days	-Resolution of CMV DNAemia in 3 weeks. -Complete resolution of pneumonia in 12 weeks.
6	M	1	SAA	Haplo (father)	D + /R+	29 days	CMV pneumonia	8.48 × 10^2^ (plasma), CMV DNA in BAL fluid confirmed by NGS	Fungal meningitis, bacterial enteritis	III	-	ATG	PTCY + FK506 + MMF + LDMTX	>99%	GCV + CMV-IVIG	15 days	-Resolution of CMV DNAemia in 4 weeks. -Complete resolution of pneumonia in 6 weeks.
7	F	5	TM	Haplo (mother)	D + /R+	45 days	CMV pneumonia	4.55 × 10^2^ (plasma), 9.35 × 10^3^ (BAL fluid)	EBV DNAemia, fungal pneumonia	II	-	ATG	PTCY + FK506 + MMF + LDMTX	60–80% for the first month then >99% after 2 months.	GCV + CMV-IVIG	20 days	-Resolution of CMV DNAemia in 3 weeks. -Complete resolution of pneumonia in 6 weeks.
8	F	5	TM	Haplo (father)	D + /R+	40 days	CMV pneumonia	7.14 × 10^2^ (plasma), 2.48 × 10^2^ (BAL fluid)	-	IV	-	ATG	PTCY + FK506 + MMF + LDMTX	>99%	GCV + CMV-IVIG	21 days	-Resolution of CMV DNAemia in 3 weeks. -Complete resolution of pneumonia in 5 weeks.
9	F	4	TM	Haplo (sister)	D + /R+	33 days	Refractory CMV DNAemia	5.78 × 10^3^ (plasma)	EBV DNAemia	II	-	ATG	PTCY + CSA + MMF + LDMTX	<50% for the first months then >99% after 2 months.	GCV + CMV-IVIG	19 days	Resolution of CMV DNAemia in 2 weeks.
10	M	2	SAA	Haplo (father)	D + /R+	27 days	CMV pneumonia	1.33 × 10^3^ (plasma), 1.35 × 10^3^ (BAL fluid)	Invasive fungal infection, adenoviral enteritis	-	-	ATG	PTCY + CSA + MMF + LDMTX	>99%	GCV + FOS + CMV-IVIG	23 days	-Resolution of CMV DNAemia in 4 weeks. -Complete resolution of pneumonia in 5 weeks.

*ATG* anti-thymocyte globulin, *BAL fluid* bronchoalveolar lavage fluid, *CMV* cytomegalovirus, *CSA* cyclosporine, *CMV-CTLs* cytomegalovirus specific cytotoxic T lymphocytes, *D* donor, *F* female, *FK506* tacrolimus, *FOS* foscarnet, *GCV* ganciclovir, *Haplo* haploidentical, *HBV* hepatitis B virus, *IVIG* intravenous immunoglobulin, *LDMTX* low dose methotrexate, *M* male, *MMF* mycophenolate mofetile, *MSD* matched sibling donor, *NGS* next-generation sequencing, *PTCY* post-transplant cyclophosphamide, *PVB19* parvovirus B19, *R* recipient, *RSV* respiratory syncytial virus, *SAA* severe aplastic anemia, *TM* thalassemia major.

CMV serostatuses were D + /R + and D + /R− in 7 and 3 patients, respectively. CMV infection developed within the first 2 months after transplantation in all patients (median 39 days, range 27–63 days). Severe CMV pneumonia was observed in nine patients, one of whom also developed CMV retinitis. The remaining patient had persistent CMV DNAemia. All patients followed a standard anti-CMV treatment strategy consisting of intravenous ganciclovir with or without foscarnet [3]. Eight patients received CMV-specific intravenous immunoglobulin.

All except one patient received two transfusions of CMV-CTLs at an interval of 2 weeks. The first infusion was administered at a dose of 0.5 × 10^8^ cells/kg, and the second infusion was administered approximately twice of the previous dose. Patients received the first dose of CMV-CTLs within the first month after CMV infection (median 19 days, range 12–29 days). Only one patient (patient 1) received CMV-CTLs 6 months after the first episode of CMV infection due to worsening of refractory CMV diseases. One patient (patient 2) received a single infusion of CMV-CTLs because of insufficient production of CTLs. However, the patient did not appear to have a significantly delayed resolution of CMV DNAemia or diseases compared to other patients.

In all patients, CMV DNAemia decreased within ~4 weeks (median 23 days, range 15–33 days) after the last CMV-CTL transfusion. In patients with CMV pneumonia, chest computed tomography scans showed that in all patients the lung lesions had resolved within a maximum of 12 weeks. However, one patient (patient 1) experienced blindness caused by CMV retinitis. A plausible cause of the sequelae was that this patient was unable to receive intravitreal ganciclovir therapy in combination with systemic treatment during the active retinitis stage for which might potentially stabilise or restore the best-correlated vision [5]. This patient also received steroids to treat poor graft function 4–5 months after HSCT and may decrease the effectiveness of CMV-CTLs. However, the patient did not receive any steroids during CMV infection. Compared to other patients, patient 1 also had a lower percentage of CD3^+^IFN-γ^+^ effector T cells with each infusion. Nevertheless, whether effector T cells have a dose-dependent effect on the treatment results requires further investigation.

Reconstitution of immune cells provides an important guide for the treatment and prognosis of CMV infection in HSCT recipient [6–8]. We also monitored the reconstitution of immune cells by tracking the numbers and percentages of lymphocyte subsets, including CD4^+^ and CD8^+^ T cells, B cells, and NK cells in the peripheral blood of patients at selected time points (1, 2, 3, 6, and 12 months after HSCT). CD8^+^ T cells have been extensively examined for their vital role in CMV infection [6, 7, 9]. There was a significant increase in CD8^+^ T cells in most patients after CTL transfusion except in patient 1. This can help to clear an active viral infection faster and shorten the duration of treatment, as observed in other studies. CD4^+^ T cells are also important in the adaptive immune response against CMV. CD4^+^ T cells is required to facilitate and maintain the classic cytotoxic response mediated by the CD8^+^ T cell response after adoptive transfer of CMV CTLs in HSCT patients [10]. In children, impaired CD4^+^ T cell immunity has been shown to be associated with delayed regression of CMV-related diseases. However, no correlation was found between CD4^+^ T cell level and the severity of CMV diseases [11]. In this study, each CTL product contained 1–9.1% of CD3^+^CD4^+^ T cells. However, significant increases in CD4^+^ T cells were only observed in two patients (patients 8 and 9) after CTL transfusion. Instead, a steady increase in CD4^+^ T cells was observed over time in most patients. CD4^+^ T cells have been suggested to be important for the detection of CMV during latency and can therefore offer protection against late CMV infection [11]. Another interesting finding from this study is that adoptive transfer of CMV-CTLs also facilitates the recovery of non-T cell populations, including B cells and NK cells, in some patients, suggesting that CTLs may offer universal benefits for immune reconstitution after HSCT. However, the exact mechanism requires further investigation.

A major concern with the use of CMV-CTLs is the potential to develop or extravate aGVHD due to alloreactivities caused by CTLs. In the current study, aGVHD was observed in four patients at the time of CMV infection, of which only two patients had grade III–IV aGVHD. To prevent the development or aggravate of aGVHD, patients received a lower dose of CMV-CTLs on the first infusion and a higher dose on the second infusion. In accordance with other published studies, we did not observe any increase in the severity of aGVHD after CMV-CTL transfusion [12]. However, an optimisation of the dose of CMV-CTLs for paediatric patients is still required in adequately larger, randomised and prospective studies.

In conclusion, our results demonstrated that adoptive transfusion of donor-derived CMV-specific CTLs is effective and safe for the treatment of CMV diseases and refractory CMV infection in paediatric HSCT recipients. Early intervention with CMV-CTLs combined with standard antiviral treatment can facilitate immune recovery, resolve CMV diseases, and prevent latent CMV infection.
